# Supplementary material for: Development of a new perceived injustice scale for Bangla speaking population
Source: PLoS One. 2024 Oct 3;19(10):e0311272. doi: 10.1371/journal.pone.0311272 (PMC11449271; doi:10.1371/journal.pone.0311272)
Supplement: S1 File — (PDF) [file pone.0311272.s001.pdf]

Mourin Perceived Injustice Scale

নির্দেশনা

জীবনের নানা অভিজ্ঞতা আমাদের মধ্যে অবিচারের (Injustice) অনুভূতি তৈরি করে। নিচে তেমন কিছু বিষয় দেয়া আছে। এমন ক্ষেত্রে আপনার মধ্যে অবিচারের অনুভূতি তৈরি হয় কিনা অথবা এ বিষয়গুলো কতটুকু হয় তা বিষয়/বিবৃতিগুলোর ডান পাশের ৫টি ঘরের যে কোন একটিতে টিক চিহ্ন (√) দিয়ে নির্দেশ করুন।

অনুগ্রহ করে লক্ষ্য করুন সবগুলো বিবৃতির উত্তর দিয়েছেন কিনা।

		০	১	২	৩	৪
১	যখন আমাকে আমার প্রাপ্য সুযোগ-সুবিধা থেকে বঞ্চিত করা হয়।	কোন অবিচারের অনুভূতি হয় না	খুবই কম হয়	মোটামুটি হয়	অনেকটা হয়	ভীষন অবিচারের অনুভূতি হয়
২	যখন আমার যোগ্যতা অনুসারে আমাকে মূল্যায়ন করা হয় না।	কোন অবিচারের অনুভূতি হয় না	খুবই কম হয়	মোটামুটি হয়	অনেকটা হয়	ভীষন অবিচারের অনুভূতি হয়
৩	যখন পারিপার্শ্বিকতা বা সিস্টেমের বাধার কারনে আমার যা যা করতে পারার কথা তা পারি না।	কোন অবিচারের অনুভূতি হয় না	খুবই কম হয়	মোটামুটি হয়	অনেকটা হয়	ভীষন অবিচারের অনুভূতি হয়
৪	যখন ভাল/সঠিক হওয়া সত্ত্বেও আমার কাজকে মূল্যায়ন করা হয় না।	কোন অবিচারের অনুভূতি হয় না	খুবই কম হয়	মোটামুটি হয়	অনেকটা হয়	ভীষন অবিচারের অনুভূতি হয়
৫	যখন আমার সাথে অসম আচরণ করা হয়।	কোন অবিচারের অনুভূতি হয় না	খুবই কম হয়	মোটামুটি হয়	অনেকটা হয়	ভীষন অবিচারের অনুভূতি হয়
৬	যখন সামাজিক অবস্থানের কারণে আমাকে অবমূল্যায়ন বা হেয় করা হয়।	কোন অবিচারের অনুভূতি হয় না	খুবই কম হয়	মোটামুটি হয়	অনেকটা হয়	ভীষন অবিচারের অনুভূতি হয়
৭	যখন আমাকে আমার মতামত প্রকাশ করতে দেয়া হয় না।	কোন অবিচারের অনুভূতি হয় না	খুবই কম হয়	মোটামুটি হয়	অনেকটা হয়	ভীষন অবিচারের অনুভূতি হয়
৮	যখন আমাকে আমার অধিকার থেকে বঞ্চিত করা হয়।	কোন অবিচারের অনুভূতি হয় না	খুবই কম হয়	মোটামুটি হয়	অনেকটা হয়	ভীষন অবিচারের অনুভূতি হয়
৯	আমার সাথে অবিচার করা হয়।	কখনোই না	খুব কম	মাঝে মাঝে	প্রায়ই	সবসময়ই
১০	অবিচারের শিকার হলে নিজেকে খুব অসহায় মনে হয়।	কখনোই না	খুব কম	মাঝে মাঝে	প্রায়ই	সবসময়ই
