# Supplementary material for: Development of a new perceived injustice scale for Bangla speaking population
Source: PLoS One. 2024 Oct 3;19(10):e0311272. doi: 10.1371/journal.pone.0311272 (PMC11449271; doi:10.1371/journal.pone.0311272)
Supplement: S2 File — (PDF) [file pone.0311272.s002.pdf]

Mourin Perceived Injustice Scale (English Translation)

Instructions.

Our experiences in life often give us feeling of injustice. A few of such experiences are presented below. Read carefully and check based on the right side responses, how much these situations applies to you. Please mark (✓) your response in one of the 5 boxes as it matches with your experience.

Please check if you have responded to all the items.

		0	1	2	3	4
1	When I am being deprived of the facilities that I deserve.	Do not feel any injustice	Very little	To some extent	To a great extent	Feel too much injustice
2	When I am not valued as per my qualification.	Do not feel any injustice	Very little	To some extent	To a great extent	Feel too much injustice
3	When contextual or systemic barriers prevent me from doing what I could do.	Do not feel any injustice	Very little	To some extent	To a great extent	Feel too much injustice
4	When despite being correct/good, my deeds are not validated by others.	Do not feel any injustice	Very little	To some extent	To a great extent	Feel too much injustice
5	When I am being treated unfairly.	Do not feel any injustice	Very little	To some extent	To a great extent	Feel too much injustice
6	When I am belittled or devalued due to my social status.	Do not feel any injustice	Very little	To some extent	To a great extent	Feel too much injustice
7	When I am prevented from expressing my opinion.	Do not feel any injustice	Very little	To some extent	To a great extent	Feel too much injustice
8	When I am being deprived of my rights.	Do not feel any injustice	Very little	To some extent	To a great extent	Feel too much injustice
9	I am treated with injustice.	Never	Very rarely	Sometim es	Often	Always
10	I feel helpless when I am being victim of injustice.	Never	Very rarely	Sometim es	Often	Always
