# Supplementary material for: Development of a new perceived injustice scale for Bangla speaking population
Source: PLoS One. 2024 Oct 3;19(10):e0311272. doi: 10.1371/journal.pone.0311272 (PMC11449271; doi:10.1371/journal.pone.0311272)
Supplement: Attachment — Submitted filename: Review comment.docx [file pone.0311272.s003.docx]

**Review comment**

The authors present the development process of a new type of psychological scale and expound on a relatively new psychological concept - perceived injustice. However, the article still requires further modification.

1. In the “background“ section or ”discussion“ section, the author should elaborate on what the specific applied values of "perceived injustice" and the "perceived injustice scale" are in clinical practice and psychological practice. Perceived injustice seems to hold a more significant position in social activities but has less evident roles in clinical applications. The author needs to further argue this point to alleviate the reader’s doubts.

2. The author mentions in the manuscript that there have been previous studies reporting scales related to 'perceived injustice'. Therefore, it is necessary to further elaborate on the similarities and differences between the old and new scales, as well as their respective advantages and disadvantages in practical applications.

3. When the Cronbach's alpha coefficient is excessively high, particularly above 0.95, it may indicate that the items in the scale are overly similar, leading to redundancy. In other words, this suggests that the scale may contain multiple items that measure the same issue, increasing the length of the scale without adding substantial information, thus reducing the efficiency of the assessment. Moreover, an overly high Cronbach's alpha may result from an overestimation of the inter-item correlations, leading to an overly optimistic appraisal of reliability. In the manuscript, the author reports a Cronbach's alpha of 0.971. A deeper analysis of the reasons behind this excessively high coefficient, the accuracy of the data, and considerations regarding whether some items should be removed is warranted.

4. In the “Participants“ section, the author estimated a minimum sample size of 226 participants, however, the actual effective sample size was merely 221, indicating that the sample size did not meet the projected requirement. Furthermore, the manuscript does not mention how many potential participants the researchers approached, how many declined to participate in the study, and how many were excluded and for what reasons.
